# Solitary Spinal Epidural Metastasis from Gastric Cancer

**DOI:** 10.1155/2016/1591269

**Published:** 2016-09-14

**Authors:** Taisei Sako, Yasuaki Iida, Yuichirou Yokoyama, Shintaro Tsuge, Keiji Hasegawa, Akihito Wada, Tetsuo Mikami, Hiroshi Takahashi

**Affiliations:** ^1^Department of Orthopaedic Surgery, Toho University School of Medicine, Tokyo, Japan; ^2^Department of Pathology, Toho University School of Medicine, Tokyo, Japan

## Abstract

Solitary epidural space metastasis of a malignant tumor is rare. We encountered a 79-year-old male patient with solitary metastatic epidural tumor who developed paraplegia and dysuria. The patient had undergone total gastrectomy for gastric cancer followed by chemotherapy 8 months priorly. The whole body was examined for suspected metastatic spinal tumor, but no metastases of the spine or important organs were observed, and a solitary mass was present in the thoracic spinal epidural space. The mass was excised for diagnosis and treatment and was histopathologically diagnosed as metastasis from gastric cancer. No solitary metastatic epidural tumor from gastric cancer has been reported in English. Among the Japanese, 3 cases have been reported, in which the outcome was poor in all cases and no definite diagnosis could be made before surgery in any case. Our patient developed concomitant pneumonia after surgery and died shortly after the surgery. When a patient has a past medical history of malignant tumor, the possibility of a solitary metastatic tumor in the epidural space should be considered.

## 1. Introduction

In most cases of metastatic epidural tumor the tumor expanding from a vertebral metastasis grows into the spinal canal, and solitary epidural metastasis of a malignant tumor is rare. We encountered a patient with a solitary metastatic epidural tumor from gastric cancer and with no vertebral metastasis.

## 2. Case Report

The patient was a 79-year-old man with the chief complaints of paraplegia and dysuria.

Bilateral muscle weakness of the lower limbs developed with no known cause in October 20××. The symptoms gradually became aggravated, and it became difficult for the patient to stand up from November. Dysuria appeared after several days and activities of daily living also became difficult. Thus, the patient was urgently admitted for close examination and treatment.

The patient had a past medical history of gastric cancer and had undergone total gastrectomy in March, followed by postoperative chemotherapy (combination of oral 5FU preparation S-1 + cisplatin) from April to July.

The muscle strength of the lower limbs on admission was MMT3 in all of the bilateral iliopsoas, quadriceps, tibialis anterior, extensor hallucis longus, and flexor hallucis longus muscles, showing complete bilateral muscle weakness of the lower limbs. Regarding sensation, 1/10 hypaesthesia was noted in the T4 or lower regions. The bilateral patellar tendon and Achilles tendon reflexes were normal, and Babinski and Chaddock reflexes were negative. Since difficulty in urination and abdominal distension were noted, urethral catheterization was performed, and 1400 mL of urine was drained.

On blood testing, WBC was 4,200/*μ*L and CRP was 0.6 mg/dL, showing a mild inflammatory response, Hgb was 9.9 g/dL, and Plt was 10.9 × 10^4^ *μ*L, showing pancytopenia. LDH was 275 IU/L, AST was 33 IU/L, and ALT was 59 IU/L, indicating elevation of liver enzymes. ALP was 355 IU/L, and Ca was 8.0 mg/dL. With respect to tumor markers, CEA, CA19-9, and AFP were increased to 15.2 ng/mL, 1,420 U/mL, and 1,034 ng/mL, respectively.

On cerebrospinal fluid testing, the fluid was transparent, and the cell count was elevated to 32/*μ*L (polymorphonuclear leukocyte: 81%, mononuclear cell: 19%). The glucose and protein levels were increased to 104 mg/dL and 181 mg/dL, respectively. CEA was <0.50 ng/dL, which was within the normal range. No malignant findings were noted on cerebrospinal fluid cytology, which was judged as Class I.

**Figure 1:**
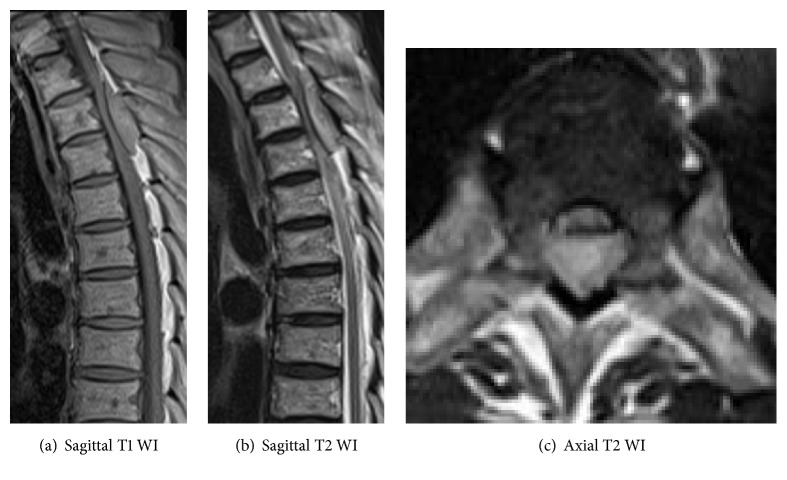
Thoracic spine MRI before operation. A thoracic epidural mass lesion was evident showing low intensity on the T1 weighted image and iso-high intensity on the T2 weighted image.

**Figure 2:**
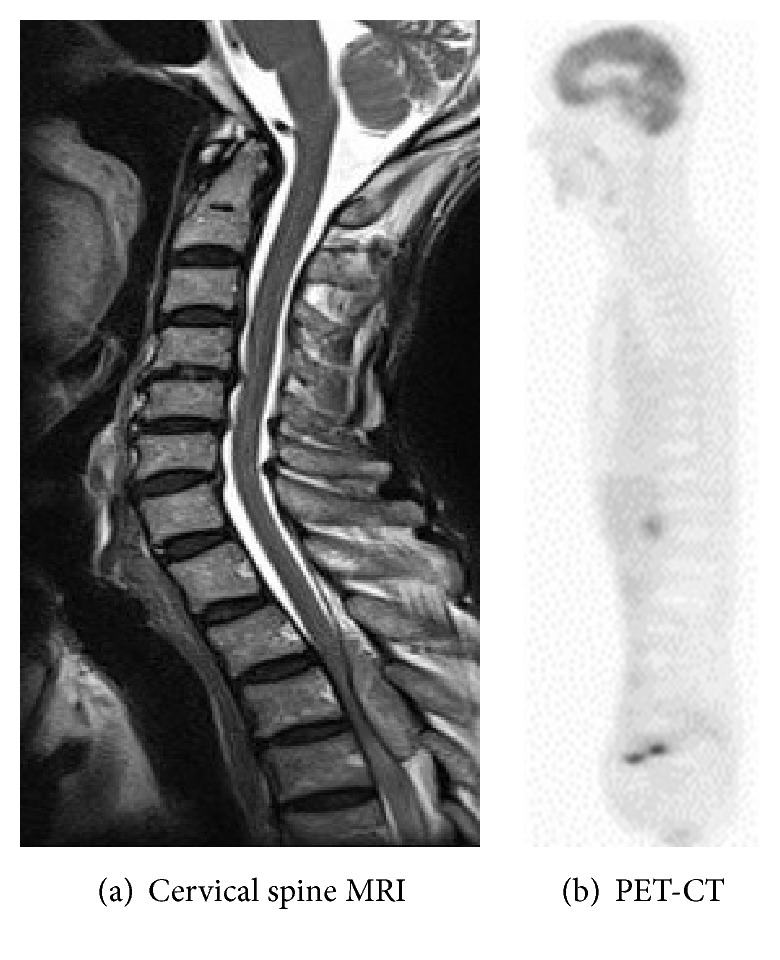
No apparent mass lesions other than the thoracic spinal epidural mass were observed.

On thoracic spinal plain radiography, no apparent abnormal findings, that is, osteolytic findings of the vertebrae and pedicle sign, were noted. On thoracic spinal MRI, a low-isointensity region was observed in the dorsal dura mater on T1-weighted imaging and an iso-high intensity mass lesion was observed on T2-weighted imaging at the T2–4 level; the lesion excluded the dural canal from the dorsal side (Figure 1). On head, chest, and abdominal CT, head and spinal MRI, and PET, no apparent mass lesions other than the thoracic spinal epidural mass were observed (Figure 2).

Paralysis progressed from Frankel C to A at 4 days after admission, and emergency surgery was performed to treat the epidural occupying lesion of the thoracic spine. Laminectomy and tumor excision were applied at the T2–5 level. A grayish white tumorous lesion was present in the dorsal dura mater and markedly obstructed the dural canal from the posterior side. The tumor parenchymal tissue was fragile and hemorrhagic. Since adhesion between the dura mater and tumor was marked, en bloc excision was attempted but was difficult, and the lesion was resected piece by piece as much as possible, and sufficient decompression of the dural canal was achieved.

**Figure 3:**
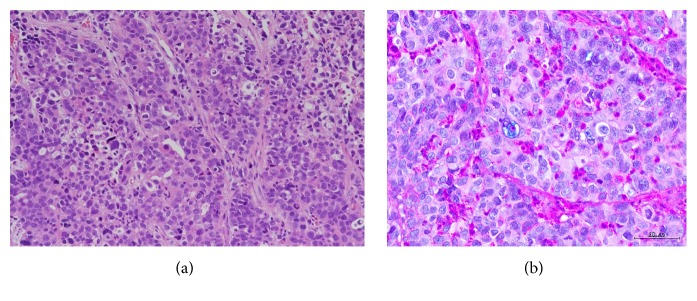
(a) Hematoxylin and eosin (HE) staining showed outgrowth of cells containing swollen nuclei to a solid tumor which was observed, with a few gland duct-like structures (×400). (b) Alcian-blue staining-positive mucus production was observed, suggesting poorly differentiated adenocarcinoma (×400).

On pathological examination, outgrowth of cells containing swollen nuclei to a solid tumor was observed, with a few gland duct-like structures. Alcian-blue staining-positive mucus production was observed, suggesting poorly differentiated adenocarcinoma (Figure 3). The histology of the lesion was similar to that of lymph node metastasis, which is observed in gastric cancer surgery, suggesting metastasis from gastric cancer.

The neurologic manifestation did not improve after surgery. The lesion was clinically diagnosed as metastatic tumor of gastric cancer, and treatment with irradiation and chemotherapy were suggested; however, best supportive care was selected because the general condition was poor due to the complication with pneumonia. Ultimately, pneumonia-aggravated, disseminated intravascular coagulation (DIC) occurred, and the patient died 27 days after admission.

## 3. Discussion

When a patient with a past medical history of a malignant tumor develops spinal paralysis during the course of treatment, metastatic spinal tumor-associated paralysis should be the primary suspected etiology.

Since our patient had a past medical history of malignant tumor, head, chest, and abdominal CT, head and spinal MRI, and PET were performed in consideration of spinal metastasis. However, no apparent mass lesion, other than the thoracic spinal epidural mass, was noted. The sensitivities and specificities of MRI and PET for metastatic bone tumor screening are high, and those of MRI have been reported to be 91 and 95%, respectively, compared with 90 and 97%, respectively, for PET [1, 2]. Furthermore, the cerebrospinal fluid cytology finding was Class 1, indicating that this case was not a metastatic vertebral tumor but that it was likely to be a hematoma, abscess, or an epidural lesion. After admission, the paralysis worsened rapidly, and surgery was performed to local regions or via the cerebrospinal fluid. The pathological findings of the excised specimen did not contradict the features of metastasis from gastric cancer, and it was diagnosed as a solitary metastatic epidural tumor from gastric cancer.

Regarding the route of metastasis to the spine, Batson [3] proposed that an epidural internal vertebral venous plexus and a vertebral venous plexus distributing in the vertebra communicate closely and that slow blood flow transports cancer cells allowing them to implant in the spine. In other local metastatic routes, tumor cells infiltrate directly from local regions or reach via the cerebrospinal fluid; although this is not common [4]. In our patient, the route was not infiltration through spinal metastasis, but it was assumed to be solitary metastasis to the epidural space via the Batson venous plexus.

While there have been no English language reports on solitary metastatic epidural tumor from gastric cancer in PubMed, 3 cases, excluding the current case, have been reported in Japan [5–7].

No definite diagnosis was made before surgery in any of these reported cases, and laminectomy and tumor resection were performed without radiotherapy. The survival time was short (from < one month to about 5 months) (Table 1).

Metastatic epidural spinal cord compression (MESCC) was first reported by Spiller in 1925 [8]. Metastatic cancer lesions typically develop in the vertebra or epidural space and induce secondary spinal cord compression. Regarding the mechanism of the pathogenesis, tumor cells cause vertebral metastasis through the circulation in the early phase and reach the spinal cord via an indirect route in about 85% of cases [9, 10], and the paravertebral tumor grows into the spinal canal through the intervertebral foramen leading to direct compression of the spinal cord in about 15% of all cases [9, 11]. Our case exhibited spinal cord paralysis, which was induced by solitary epidural metastasis, and its development did not occur through either mechanism of MESCC. MESCC has been reported to be a common complication that develops in 2.5–5% of terminal cancer patients within 2 years before death [12–14] and the annual incidence has been reported to be 3.4% among hospitalized patients [15]. Gastric cancer patients accounted for 1-2% of patients who developed MESCC [1, 16, 17], thus, a relatively low rate. In our patient, MESCC was due to the solitary metastatic epidural tumor from gastric cancer, which could be considered very rare.

Rades et al. [17] scored the survival rate of 29 gastric cancer-associated MESCC patients, based on clinical factors, and established an index to predict outcome. Specifically, Rades et al. investigated vertebral body metastasis-induced spinal cord compression. The four cases reported in Japan, including our patient, exhibited spinal cord paralysis induced by solitary metastatic epidural tumor, and the pathogenetic mechanism was different from that of MESCC. However, when our patient was scored, one item, rapidity of developing weakness of legs, was met and the 6-month survival rate was predicted to be 20%. Thus, although the pathogenetic mechanism is different, the survival predicting score developed for gastric cancer patients by Rades et al. may also be applicable for this disease.

We were not able to make a definite diagnosis before surgery, just as it was not previously possible in cases of solitary metastatic epidural tumor from gastric cancer. Surgery was performed to improve the progression of paralysis, but if it could have been diagnosed definitively, avoidance of surgery would have been an option because of the prediction of a poor outcome. In the light of our experience with this patient, when a similar case is encountered, the applicability of surgery should be investigated after obtaining sufficient informed consent.

## 4. Conclusion

We encountered a patient with a solitary metastatic epidural tumor from gastric cancer. Although it is difficult to make a definite diagnosis, this tumor should be kept in mind to be included in the differential diagnosis when surgery is selected.

## Figures and Tables

**Table 1:** Prior case report of solitary spinal epidural metastasis from gastric cancer in Japan.

Author	Year	Age	Gender	Levels	Postoperative survival period
Yoshikawa [5]	1960	73	Female	T4-5	1 M
Yamaguchi and Usumoto [6]	1965	36	Male	L5	2 M
Sato et al. [7]	1990	58	Male	L3	5 M
Our case	2015	79	Male	T2–4	<1 M
